# Does Cardiac Physical Exam Teaching Using a Cardiac Simulator Improve Medical Students’ Diagnostic Skills?

**DOI:** 10.7759/cureus.4610

**Published:** 2019-05-07

**Authors:** Nadine Gauthier, Christopher Johnson, Ellamae Stadnick, Marissa Keenan, Timothy Wood, Michael Sostok, Susan Humphrey-Murto

**Affiliations:** 1 Cardiology, The Ottawa Hospital, University of Ottawa, Ottawa, CAN; 2 Cardiology, The Ottawa Heart Institute, University of Ottawa, Ottawa, CAN; 3 Internal Medicine - Rheumatology, The Ottawa Hospital, University of Ottawa, Ottawa, CAN; 4 Medical Education and Simulation, The Ottawa Hospital, University of Ottawa, Ottawa, CAN; 5 Medical Education and Simulation, University of Cincinnati and Cincinnati Children's Hospital, Cincinnati, USA

**Keywords:** cardiac physical exam, cardiac simulator, undergraduate physical exam skills

## Abstract

Background

Challenges in bedside teaching may be overcome by the use of high-fidelity simulators for teaching the cardiac physical exam. The purpose of this study is to compare the ability of first-year medical students (MS1) to perform a cardiac physical exam and make the correct diagnosis after instruction using standardized patients (SPs) as compared to a cardiac simulator (Harvey, Laerdal Medical Corporation, NY, US).

Methods

Thirty-two MS1 were randomized to a teaching module on either SPs or Harvey. Their performance and ability to make the correct diagnosis were evaluated during a posttest objective structured clinical examination (OSCE) on real patients.

Results

No difference in the mean OSCE score was observed (SP: M=62.2% vs. Harvey: M=57.2%, p=0.32). The SP group obtained a higher frequency of correct diagnoses (M=61.5% SP vs. M=21.0% Harvey, p=0.03). Student feedback revealed that Harvey offered superior clinical findings; however, 34.4% of students requested a combination of teaching modalities as opposed to either method alone.

Conclusions

Performance in examination skills did not differ between the SP and Harvey groups but the SP group demonstrated an improved ability to arrive at a unifying diagnosis. A combined teaching program may be ideal for transferability to patients.

## Introduction

Competent physicians must be skilled in performing a physical examination because it is an essential component of patient care despite increasing access to instrumental cardiac diagnosis [1-3]. Poor clinical skills may be a result of relying on organ-based diagnoses, dependence on imaging technology, and inadequate training at the bedside [4-5]. The emergence of simulation in medical education provides a promising alternative means of training physicians in important clinical skills [6]. Despite the importance of the physical examination, it is reported that trainees do not acquire sufficient competence in the physical exam. Another study in the late 1990s reported a lack of physical examination proficiency amongst medical students and residents, with no significant increase in competency over years of training. A study from Mangione tested the proficiency of the physical diagnosis of different learners (medical students, internal medicine residents, and emergency medicine residents) using a multimedia questionnaire. Significant deficiencies in physical diagnostic skills were found, with a median error rate of 54%, there was no improvement over three years of clinical teaching and no difference between residents and senior medical students in the detection of physical exam findings [7-8]. The authors concluded that teaching physical diagnostic skills requires more emphasis in both medical schools and residency programs.

Another group from Duke Medical Center investigated more specifically the cardiac physical exam and measured the diagnostic accuracy of 47 pediatric residents while performing a cardiac physical exam on a cardiac simulator (Harvey) using five common cardiac conditions. The mean accuracy score of the residents was 33%. Although there was a correlation between improved performance and additional training, this was not statistically significant. Poor identification of cardiac findings and low performance of family medicine residents in focused cardiovascular physical exam skills has also been reported by Horiszny and colleagues, with a detection rate of 36% for abnormal heart sounds and murmurs [9]. These studies support a pressing need for improved clinical skills training during medical school and residency.

Multiple factors are contributing to the inadequate performance of bedside teaching among medical trainees over the last several decades [10]. Organized bedside teaching is becoming more challenging; recruitment of patients with real findings for clinical exam teaching is decreasing, as most patients are now managed in an ambulatory setting and inpatients are often too unstable or unavailable to participate in clinical teaching. In addition, clinical findings may fluctuate during the course of a patient’s treatment, which makes planning teaching sessions unpredictable. Finally, bringing patients to medical schools or hospitals from home requires administrative and logistical support, which may not be widely available.

An attractive means of increasing the quality and quantity of physical exam skills training in response to these challenges is the incorporation of simulation into the curriculum. Simulation is increasingly used to teach clinical and technical skills, and the availability of high-fidelity simulators offers many advantages as compared to recruiting patients with real findings [11-12].

Harvey, developed over 40 years ago, is a high-fidelity cardiac simulator designed to reproduce abnormal heart sounds, murmurs, clicks, rubs, and extra heart sounds. This simulation-teaching device can realistically simulate 27 common and rare cardiac conditions. Harvey has a very good physical resemblance to patients, including auditory resemblance to real heart sounds, tactile resemblance for pulses, and accurate resemblance to the chest wall for placement of the stethoscope.

The benefit of the high fidelity of Harvey has been previously described [13]. For example, Issenberg et al. were among the first investigators to show that students benefit from learning heart sounds and murmurs on a high-fidelity simulator (Harvey). Medical students experienced a 33% increase in their ability to accurately recognize abnormal findings on a physical exam after a two-week Harvey elective compared to only a 5% increase in accuracy in the control group exposed to standard bedside teaching when tested on Harvey [13-14]. Although studies have examined the use of Harvey as a teaching tool, these studies are small and limited by several methodological issues and most were not designed to test for the transferability of skills learned on a simulator to real patients. For example, some studies simply compared the ability of students to identify heart sounds after training on Harvey as compared to training with an audio CD [15]. Perhaps, not surprisingly, no difference was found.

It is not clear how well students taught on Harvey can transfer cardiac physical examination skills to real patients. Transfer of learning is defined as the application of knowledge and skills learned in one context to another [15]. One study that used a larger number of students and measured transferability is the study by Ewy and colleagues. They followed the performance of 208 senior medical students at five American medical schools [15]. Fourth-year medical students who used Harvey during their cardiology elective performed significantly better than the non-cardiopulmonary simulator-trained group, who learned in the traditional manner from real patients. The students were subjected to a real patient post-test with significant improvement (p<0.001) in their clinical skills, demonstrating that performance on Harvey can transfer to real patients [16]. This study did not outline the variable clinical experiences of the students and did not consider higher level skills such as diagnostic ability.

To date, no study has explored Harvey-trained students in their comprehensive proficiency in making the correct cardiac diagnosis, as opposed to simply identifying a particular heart sound when compared to students trained on SPs with real cardiac findings. A small study by Perlini and al. assesses the acquired knowledge of five common cardiac diagnoses in 42 students (MS3, MS4, and first-year Internal Medicine Residents (IM1), five years after a simulation-based tutorial without comparison to another teaching arm. After the Harvey tutorial, the most senior students (MS4 and IM1) improved their overall performance in making the correct diagnosis on Harvey (73.1% and 76.1%, p<0.001) when compared to before the tutorial [16]. Although the authors attempted to consider confounding factors, the five-year time lag between the intervention and the outcome measure make this study difficult to interpret.

This study will explore the use of Harvey to teach cardiology clinical skills to first-year medical students. Specifically, can Harvey replace the use of SPs in teaching physical exam skills and will these skills be transferable to real patients? The purpose of this study, therefore, is to compare the clinical performance of medical students taught on Harvey as compared to the standard teaching method using standardized patients (SPs) with real cardiac findings. Students will be compared on their ability to perform a cardiac physical exam, detect common heart murmurs, and correctly arrive at one unifying diagnosis based on their physical examination on real patients. A secondary outcome will be to explore student preferences for learning and similarities of clinical findings on Harvey as compared to learning on SP.

## Materials and methods

This study was approved by the Ottawa Health Science Network Research Ethics. Forty participating students provided written consent and were provided with a unique study number to guarantee anonymity.

Participants

All first-year medical students from the University of Ottawa first-year class (n= 150) were invited to participate in this study. First-year students were selected to avoid contamination, i.e., minimal previous clinical experience. Due to the limited availability of tutors and simulation equipment, the study was limited to the first 40 students who provided consent (20 students in each teaching arm). Finally, 32 students completed the five phases of the study and were included for analysis. The reasons for excluding eight students; three students in the SP group and one student in the Harvey group, was that they could not attend the post-test due to scheduling issues. In addition, due to technical issues with the video recordings during the clinical OSCE, four students in the SP arm were removed.

Randomization, baseline questionnaire

**Figure 1:**
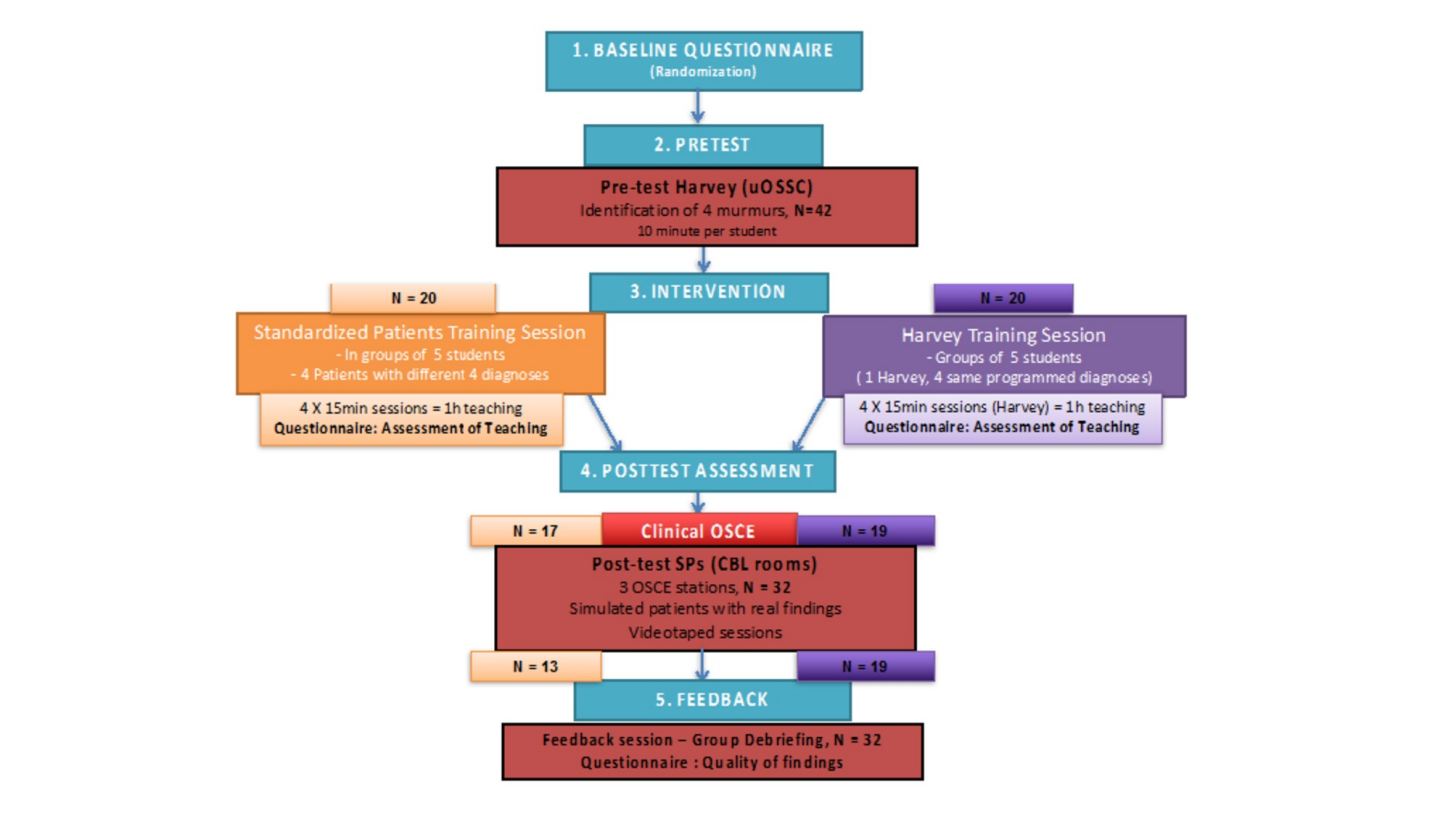
Five-phase Study Design

This study was divided into five phases, as outlined in Figure 1. In the first phase, all first-year medical students were invited to an introduction session. The principal investigator (PI) was not present to prevent potential coercion, as the PI is also the content expert of the cardiology undergraduate rotation. A short video of the Harvey simulation was presented to introduce the cardiac simulator. The first 40 students who were interested in participating were randomly allocated by an allocation software once anonymized into one of two teaching modality arms: (1) SPs with real cardiac findings group (control group) or (2) Harvey, the cardiac simulator (intervention group).

Pretest Assessment

The pretest assessment took place at the University of Ottawa Skills and Simulation Centre (uOSSC) using Harvey, the cardiac simulator. The participants were asked to review five cases during a short pretest. For each case, Harvey was programmed by one of the co-investigators to display a specific cardiac condition with relevant associated findings. The students had two minutes to auscultate the simulator followed by 30 seconds to complete a multiple-choice questionnaire asking them to identify the type of murmur as well as the associated clinical diagnosis. Participants were prompted to palpate the carotid pulse and proceed to auscultation. Assuming that first-year medical students would have minimal experience or knowledge of cardiac physical exam skills this early on in their medical training, two minutes with the simulator was deemed enough in the pretest component of the study.

Intervention

All students received the same 15-minute introduction to a cardiovascular physiology and anatomy lecture and were taught a standard approach to a focused cardiac physical examination, including carotid pulse palpation and auscultation. The second one-hour session was different depending on randomization. All were instructed on the identification of four common cardiac conditions: aortic stenosis (AS), aortic regurgitation (AR), mitral regurgitation (MR), and tricuspid regurgitation (TR). This was completed in four sequential, 15-minute sessions, one session for each diagnosis for a total of one hour of hands-on teaching. Each student had the chance to practice a focused physical examination at least once for every cardiac diagnosis during this allocated session. The only difference was the platform used for teaching; the control group received core cardiac physical exam training on SPs with real findings and the intervention group received the same physical exam training on Harvey, the cardiac simulator. Two clinical cardiologists with at least three years' teaching experience provided teaching on both SPs and Harvey and taught the same content. Each teacher was assigned four groups: two groups with teaching on SPs, two with teaching on Harvey. Each group contained five students, respectively, providing standardized teaching to a total of 20 students.

Each 15-minute session focused on one diagnosis. The teaching platform varied between groups. In the control group, SPs with real physical findings were examined and assessed by the two cardiologist teachers one week prior to the teaching session. The cardiologists examined each patient in order to compare and quantify the perceived level of difficulty in identifying the clinical findings. For the intervention group, Harvey was programmed to produce each of the four conditions. Harvey is capable of producing heart sounds, including murmurs, variation in heart sounds, extra heart sounds, and changes in pulses as appropriate.

Immediately following the teaching sessions, the participants were asked to complete a five-item questionnaire rating their learning experience. The students were asked to select their preferred teaching platform; the students in the Harvey teaching arm were asked if they would have preferred physical exam teaching on an SP with findings and the students in the SP teaching arm were asked if they would have preferred teaching on Harvey.

Post-test Assessment

The primary outcome was measured with the objective structured clinical examination (OSCE) using real patients with three clinical diagnoses, which were taught during the intervention; therefore, no new content was assessed. The students had a total of seven minutes with each patient to complete a focused cardiac physical examination (palpate carotid pulse and cardiac auscultation in four designated areas on the chest wall). The three chosen conditions for assessment on real patients included aortic stenosis, mitral regurgitation, and a real patient with normal cardiac findings for standardization. The sessions were videotaped for future viewing by two other expert cardiologists trained as examiners who were blinded to group randomization. Similar to the teaching intervention, these real post-test patients were assessed by the original teachers immediately before the assessment in order to document the perceived difficulty level of detecting and correctly identifying these physical findings.

An OSCE checklist was developed by the principal investigator and consisted of 13 items. Items were then reviewed by one other expert cardiologist who participated in the teaching curriculum. Three of the 13 items were specific for the identification of abnormal physical exam findings. One item asked if the student “identified a murmur,” a second if they could “differentiate a systolic from a diastolic murmur,” and, lastly, if the student could “identify the correct diagnosis.” For each item, examiners were asked if the student “performed correctly” (1 point) or “performed incorrectly/not attempted” (0 points). Each video was viewed once by one cardiologist who then completed the checklist. Each cardiologist viewed 48 OSCE recordings. In order to allow consistency in rating, one examiner reviewed all the “aortic stenosis” stations, the other reviewed all the “mitral regurgitation” stations, and they shared the “normal findings” station.

Feedback

The participants were then invited to a group debriefing session to share their general impression and feedback of the study as well as to review the post-test clinical diagnoses for additional learning. At the end of the one-hour teaching session, the students completed a short survey regarding the perceived differences of the cardiac findings on the teaching platforms (SP or Harvey) and the OSCE post-test SP.

## Results

Quantitative data

From a population of 150 first-year medical students at the University of Ottawa, 40 students provided consent and 32 students completed the study, as explained later. Students were provided with a unique study number to guarantee anonymity.

Table 1 displays baseline demographic data for the standard patient and Harvey groups. At the time of recruitment, the participants provided demographic information, such as age, gender, professional qualifications, and previous clinical experience in cardiac auscultation, prior to starting medical school.

**Table 1:** Baseline Demographics of Participating First-Year Medical Students *Chi-square p=NS

	SP Group (n =13)	Harvey (n=19)
Number of participants	13	19
Male	2 (15%)	10 (53%)
Female	11 (85%)	9 (47%)
Academic degrees		
Undergraduate sciences	5 (39%)	9 (47%)
Undergraduate health sciences	6 (46%)	8 (42%)
Graduate studies	2 (15%)	2 (11%)
Hours in clinical setting	3.1	3.5
Previous use of stethoscope	4 (31%)	7 (37%)
Hours listened to heart sounds	1.4	1.4

There were no statistically significant differences between the groups. Roughly equal proportions of students had undergone science studies prior to medical school and reporting minimal health care or clinical training experience. There was also no difference between the two groups in terms of clinical setting exposure with an average of one and one-half hour of prior use of a stethoscope for cardiac auscultation. There were more female students in the SP group (85% vs. 47%) but this did not reach statistical significance.

Pretest

Table 2 displays the results of the total score of the MCQ pretest; the mean of the SP group (M=51%) did not differ significantly from the mean of the Harvey group (M=33%). Table 2 also displays the mean scores for these individual questions as well. For each individual score, the observed means of the standardized did not differ from the means of the Harvey group, suggesting an equal baseline level of knowledge in cardiac physical examination.

**Table 2:** Harvey Multiple-Choice Pretest Percentage Scores (%) *Average score of four pretest clinical questions in percentages, p-values were non-significant. OSCE: objective structured clinical examination

	SP (n=13)	Harvey (n=19)	p-value	Effect size
Total Pretest Score * (SD)	57 (21)	48 (23)	0.26	.41
Individual OSCE Station Scores				
Aortic Stenosis Score (SD)	51 (17)	33 (29)	0.48	0.76
Aortic Regurgitation Score (SD)	49 (35)	40 (31)	0.25	0.27
Mitral Regurgitation Score (SD)	51 (22)	42 (22)	0.28	0.41
Tricuspid Regurgitation Score (SD)	28 (25)	39 (25)	0.28	0.44

Post-test

The pretest covariate was not significantly related to the post-test scores (F (1,29)=.006, p=.94, E.S.=.00) and there was no significant difference in post-test scores between the two groups (F (1,29)=1.11, p=.30, E.S.=.04). Because the covariate was not significant, only the observed means are reported in Table 3. The SP group obtained an average of 62.2%, and in the Harvey group, an average score of 57.2%. For each OSCE station (aortic stenosis, mitral regurgitation, and normal cardiac findings), the difference between groups was not significant (aortic stenosis; 69% vs. 70%, respectively, F (1, 30)=0.072, P=0.79, E.S.=0.02, mitral regurgitation: M=57% vs. 50%, F (1, 30)=1.250, P=0.27, E.S.=0.04) and normal findings: M=60% vs. 51% F (1, 30)=1.004, P=0.32, E.S.=0.002).

**Table 3:** Real Patient Post-Test Clinical OSCE Performance Scores (%) *Total OSCE scores out of 13 expressed as a percentage, p=NS ** Individual station OSCE scores out of 13 expressed as a percentage, p=NS OSCE: objective structured clinical examination

	SP (n=13)	Harvey (n=19)	p-value	Effect size
Total OSCE score* (SD)	62.2 (12)	57.2 (13)	0.30	0.04
Individual OSCE station scores**				
Aortic stenosis (SD)	69 (10)	70 (9)	0.79	0.002
Mitral regurgitation (SD)	57 (15)	50 (17)	0.27	0.04
Normal findings (SD)	60 (28)	51 (26)	0.32	0.002

Table 4 displays the OSCE total correct diagnosis score for the two groups. Fisher’s exact test was conducted for this analysis. Before running this analysis, the groups were combined into a low correct diagnosis score (0/3 and 1/3) and a higher correct diagnosis score (2/3 and 3/3). A greater number of students in the SP group obtained a higher correct diagnosis score (61.5%) than in the Harvey group (21.0%), p=0.03.

**Table 4:** Number of Students Achieving Correct Diagnosis Scores - Post-Test Clinical OSCE * Correct diagnosis score: one point per station to a maximum of three points for whole OSCE, expressed as frequencies ** High correct diagnosis score: combined results for 2/3 and 3/3 scores Correct diagnosis score (8/13=61.5%) SP vs Harvey group (4/19=21.0%), p=0.03 OSCE: objective structured clinical examination

Correct Diagnoses Score *	SP (n=13)	Harvey (n=19)
0/3	3	6
1/3	2	9
2/3**	7	4
3/3**	1	0

Open-ended Comments

Assessment of Teaching

Table 5 displays the mean ratings for the Assessment of Teaching questionnaire. When asked to rate the overall teaching experience, the difference between the two groups was not significant (t (35)=-0.60, p=.55). The participants were equally satisfied with their tutor (t (35)=0.42, p=.68). Participants rated the quality of the heart sounds on Harvey as being superior to the SP (t (35)=-8.0, p<.000). With regards to practice time and preference, the difference between the groups was not significant for both items (t (35)=-1.25, p=0.22 and t (35)=-0.24) p=0.8).

**Table 5:** Assessment of Teaching Questionnaire - Open-Ended Comment Analysis *1=poor, 2=borderline, 3=neutral, 4=good, 5=very good, 6=excellent ** 1=definitely no, 2=not really, 3=neutral, 4=yes to some extent, 5=definitely *** 1=strongly disagree, 2=strongly agree, 3=neutral, 4=agree, 5=strongly agree

	SP (n=13)	Harvey (n=19)	p-value
Overall teaching experience* (SD)	4.17 (0.83)	4.32 (1.25)	0.55
Tutor rating* (SD)	4.92 (0.79)	4.68 (1.34)	0.68
Quality of cardiac findings* (SD)	2.92 (0.90)	5.16 (0.83)	< 0.000
Appropriate practice time** (SD)	2.83 (1.12)	3.42 (0.96)	0.22
Preference for other intervention arm*** (SD)	3.5 (0.67)	3.68 (1.16)	0.81

General Feedback

The students were asked to provide comments on their preference in terms of the teaching modality between SP and Harvey immediately after the teaching session. Five main themes were identified: “Harvey has clearer physical exam findings,” “Harvey is the preferred teaching modality,” “SPs are more realistic,” “SPs are the preferred modality of teaching,” and, finally, “A combination of both modalities is preferred.” Of the participants, 34.4% believed that a combination of both teaching modalities would be preferred as opposed to Harvey alone (9.4%) or SP alone (6.2%). Students felt Harvey provided clearer examination findings (18.7%), which improved student satisfaction, yet SPs were deemed important because they provided a more “realistic” experience and provided better patient contact (15.6%).

When asked to compare the heart sounds and murmurs of the SP or Harvey during the teaching session to the findings of the real patients during the clinical OSCE, the participants in the SP group reported a neutral relationship between the two types of patients (M=3, 3 being “neutral”) while the Harvey group commented on a greater difference between Harvey and real patients (M=4, 4 being “different,” p=0.018). Similar observations were captured with a question asking about the similarity of the carotid pulse on the SP (M=2) or Harvey in comparison to the real patients during the OSCE. (M=4), p=0.000.

Although no cross-over study was conducted, the students were exposed to both teaching modalities at one point in the study; all were introduced to Harvey during the pretest and all were tested on real patients with cardiac findings. It was, therefore, possible for them to compare their experience in the teaching of the cardiac physical exam on SP vs. Harvey and express their preferred method of teaching.

General comments were compared in both the teaching modality and the real patients in the OSCE and generated three overall themes: “SP has clearer findings,” “Harvey has clearer findings,” and “both groups are equal in terms of quality of findings.” When comparing the SPs with the real patients from the post-test OSCE, 50% of all the participants deemed the physical exam findings to be subtler in the SP in comparison to the real patients. In the Harvey group, 52% of participants believed that Harvey had clearer findings, as they were easier to detect. Several students in the Harvey group noted that learning on the simulator was generally easier, as they did not have to manage issues such as the bedside approach to a real patient, and, specifically, performing cardiac auscultation in female patients where students must deal with the fact that the breasts are located in proximity to the area where the stethoscope must be applied in order to auscultate over the cardiac apex.

The post-test session was followed by an informal verbal feedback session on their overall experience following real patient exposure in an OSCE setting, which generated four other themes on the overall experience. The following themes were identified: “the user-friendliness of Harvey in terms of clear and controlled findings,” “the uncomfortable setting of examining real patients without the guidance of a more experienced preceptor,” and “examining female patients where the breast imposes a challenge when auscultating the apex.” The majority felt that the two teaching modalities would be complementary due to the lack of realism in Harvey, the cardiac simulator that offers clear and reproducible cardiac findings.

## Discussion

The purpose of this study was to assess the ability of first-year medical students to detect common heart murmurs and to make the correct clinical diagnosis after receiving teaching on Harvey, the cardiac simulator, as compared to teaching on SPs with real cardiac findings. In this study, there was no difference in performance between the SP-taught students and the Harvey-taught students for overall scores.

Performance in this study was measured with an OSCE using real patients. Students were graded on different components of the focused cardiac physical exam, such as technique (i.e., palpate the carotid pulse, auscultate in the aortic area); identification of heart sounds and murmurs; recognition of the cardiac phase associated with the murmur (systole vs. diastole); and, lastly, commitment to a unifying clinical diagnosis. There was no difference between the teaching platforms in scores for overall OSCE performance or individual station scores, however, the SP-taught students appeared to have an improved ability to correctly arrive at a unifying diagnosis of the cardiac condition. These results suggest that there is better transferability to real patients when using an SP rather than a cardiopulmonary simulator.

Each teaching platform has advantages and disadvantages. Harvey provides more consistent abnormal findings as compared to real patients. Students in the Harvey group reported clearer findings and an easier initial exposure to cardiac pathology. During a Harvey teaching session, the students auscultated with individual electronic stethoscopes simultaneously with the teacher, making it easier for the teacher to comment on and outline abnormal findings in real time. This is in contrast to the difficulty with the SP platform for teaching, where there is variability in heart sounds and murmurs. The SPs were selected for the teaching sessions because they have real cardiac conditions and the associated abnormal physical examination findings. However, in addition to the abnormal finding selected for teaching, two of the SPs also had additional subtle findings on the particular day of the teaching session: one had faint aortic regurgitation and a second had a soft mitral regurgitation murmur. This added difficulty for the students within the SP group, as it was their first and only exposure to SPs prior to the post-test. This variability in physical examination findings is a disadvantage of using SPs for teaching cardiac physical examination.

A review of the videotaped OSCE by experienced examiners revealed a major disadvantage of using Harvey to teach physical examination skills to first-year medical students. Early in clinical training, students should learn an approach to the bedside physical examination, including the skills needed to be comfortable while correctly performing physical exam maneuvers. The examiners who rated the videotaped OSCE comment on the lack of such skill and a general discomfort among the medical students during the observed physical examination mostly when attempting to auscultate the apex of the heart in female patients. Corroborating this observation made independently by two experienced examiners, several students from the Harvey group commented on their lack of comfort and skill in auscultating the heart in women. Two of the three real patients used in the OSCE were female, and comments on the post-test questionnaire reported increased anxiety among the students related to examining female patients. The students’ discomfort when examining the female OSCE patients may have reduced their ability to correctly perform a cardiac physical examination, correctly interpret their findings, and arrive at the correct diagnosis. Students who had been taught on an SP rather than Harvey may have had an advantage, as they would have more experience in performing a physical examination on a real person. In addition, there were more male students in the Harvey group. This raises the possibility that the challenges imposed on the students by having to examine female OSCE patients may have been more problematic in the Harvey group. However, a review of comments from students indicates that female students also felt uncomfortable examining the apex in female patients.

A major strength of this study compared to previous research is that the outcome was the accuracy of the physical examination performed on the SP rather than on the Harvey simulator. This highlighted the importance of considering trade-offs between physical resemblance and functional task alignment when choosing to use a simulator versus real patients to teach physical examination. Hamstra and colleagues recently defined physical resemblance as any tactile, visual, auditory, and olfactory features of the simulator that enhance its physical appearance, previously defined as structural fidelity [17]. Functional task alignment replaces the term functional fidelity by emphasizing the importance of the functional aspects of the simulator with the context. There is evidence supporting the notion that a reduction in physical resemblance has minimal impact on the educational effectiveness if the functional aspects of the simulator correspond to the applied context [18]. The SP-trained group had the advantage of high functional task alignment (real chest wall, female patients with breasts, real clinical setting) as compared to the Harvey-trained group. Students trained on Harvey had the advantage of high auditory and tactile resemblance as compared to using SPs whose physical exam findings varied. The fact that both groups had similar performances on the OSCE suggests that the relative advantages of each teaching method canceled out any group differences, at least when tested in first-year medical students. It should be noted, however, that SP-taught students appeared to be more skilled in coming to the correct unifying diagnosis. Presumably, the high functional task alignment was an advantage for very junior trainees and more important than the physical resemblance of the simulator, as first-year medical students need to learn general bedside physical exam skills prior to the specifics of the cardiac physical exam. Junior trainees may, therefore, require the functional aspect of learning on SPs to acquire confidence and an appropriate bedside approach to physical examination. If the cardiac simulator could provide that clinical context, it could lead to an increase in educational effectiveness.

Other small studies measured an increase in the acquired self-confidence and cardiopulmonary physical examination skills of 56 and 33 physician assistant students, respectively, after didactic sessions and independent study, including practice time on Harvey. These studies shared similar outcomes in terms of reported confidence and preferred method of teaching but there was no comparison with a teaching arm using SP, which is the standard and traditional way of teaching cardiac physical examination [19-20].

This study was completed with very junior trainees. The first-year medical students had not been exposed to any cardiovascular teaching or training, as this study was conducted in their third month of medical school. With experienced trainees, the Harvey group may have been at less of a disadvantage, and perhaps the high resemblance of Harvey to cardiac pathology would have translated into superior OSCE scores relative to experienced trainees taught using SPs. In first-year medical students, Harvey may be a valuable teaching aid to reinforce physiology concepts, as students learn about heart sounds and murmurs rather than the teaching of cardiac physical exam skills with valvular pathology. Further research using a study design similar to ours in trainees of different levels is warranted in order to determine how and when medical curricula can optimally use Harvey.

Student experience was also important in order to fully assess the potential benefits of simulation at the undergraduate level. The students commented on their preference of teaching modality using either simulation or standardized teaching with patients. The quality of the teaching and the preparedness and efficacy of the teacher were highly rated by both groups. There was a statistical difference between their rating of the quality of the cardiac heart sounds and murmurs on Harvey and the SP. The students in the Harvey group were highly satisfied with the quality of the findings on Harvey while students in the SP arm believed the findings were more difficult to identify and, as a result, they felt that the SP teaching was less beneficial. The variability of cardiac findings among SPs, as documented by the two teachers and expert cardiologists at the beginning of the session, highlights the practical challenges related to the inconsistent findings associated with using SPs with real clinical findings as a teaching platform.

This study has several strengths. The SP and Harvey-trained groups were well-balanced, with no difference in pre-test scores. The time on task was consistent between the groups as was the exposure to each teacher with the same standardized content. As noted above, a major strength is the use of examination of real patients rather than a mannequin as a primary outcome.

Several limitations of the study must be acknowledged. First, the choice to use first-year medical students for participation in a cardiac physical examination study may seem questionable given their lack of knowledge and minimal experience in physical exam skills; the lack of professional and clinical exposure most likely contributed to their poor performance and lack of improvement in skills after the teaching intervention. The recruitment of participants and student drop-off during the five-phase design led to relatively low numbers in both groups, which limits the power of our study to detect a difference between groups. Due to technical issues with the video recordings during the clinical OSCE, four students in the SP arm were not considered in the results due to a lack of data. However, this represents a small number of students relative to the overall study and, as such, the results are not affected by the loss of this data.

## Conclusions

Today, medical educators face daunting challenges, such as reduced education funding, increased clinical demands, large class size, and a shortage of teachers and patients.^ ^Simulation-based education offers a potential solution to this challenge by enabling efficient, logistically feasible, and highly standardized training sessions. Our study comparing teaching modalities in the pre-clerkship student demonstrates that students prefer the clear, standardized cardiac findings of the cardiac simulator, but that a combined approach using SPs is essential for teaching first-year medical students cardiac physical examination. A combined curriculum ensures that students examine real patients in order to gain the skills needed to comfortably and professionally perform exam maneuvers while simulation exposes students to the pathology they may encounter during the physical exam of real patients. Future research should examine the optimal mix of physical examination teaching using standardized patients with real findings and a simulator and should investigate the roles of simulation and instruction using standardized patients at various levels of medical training.
